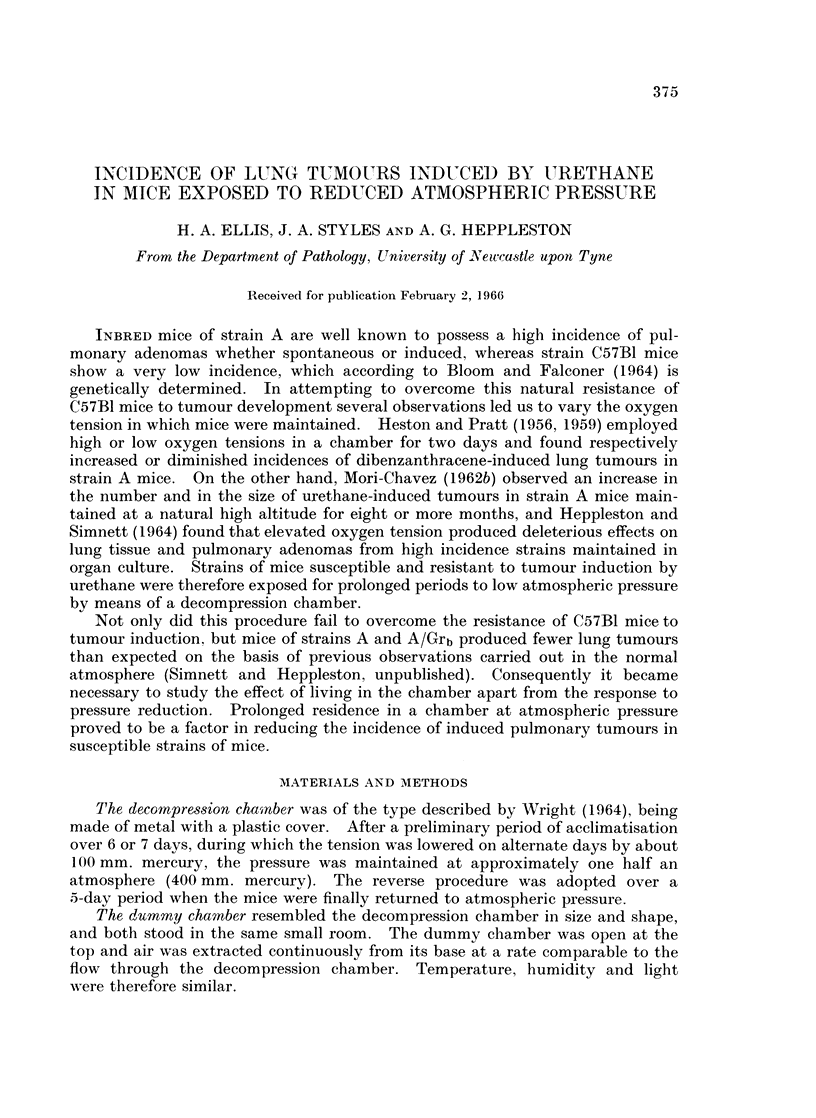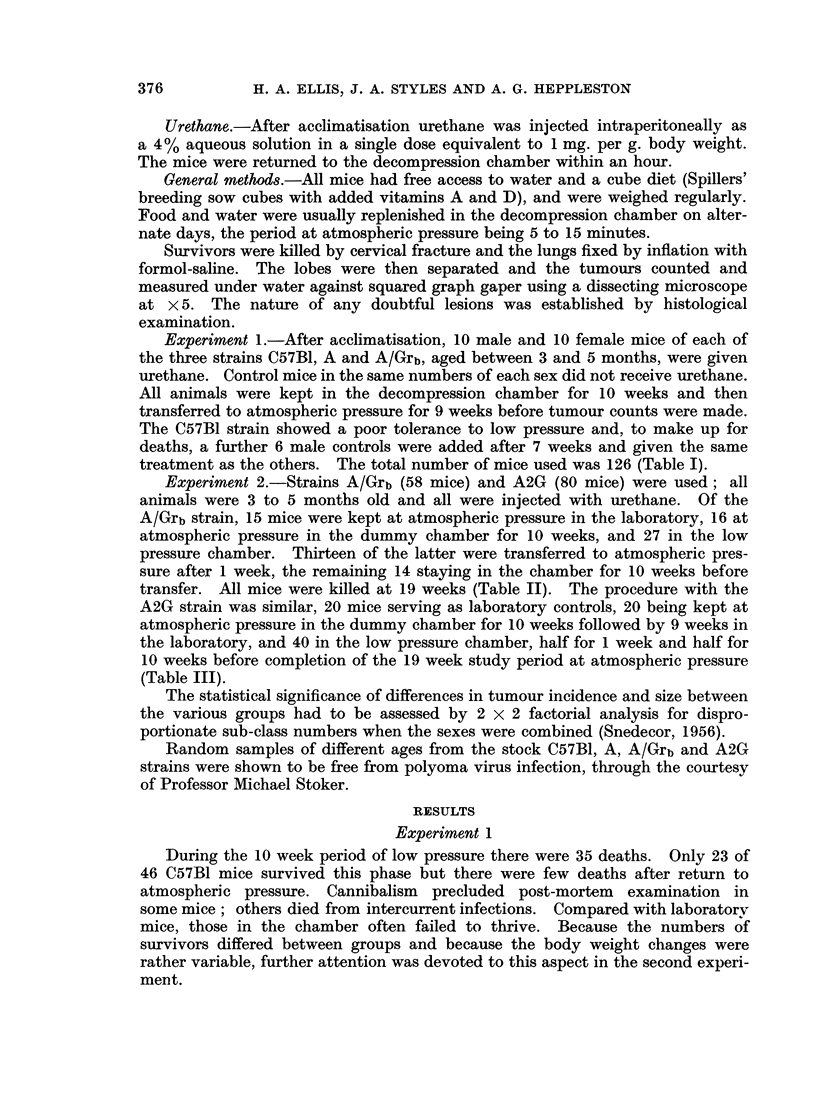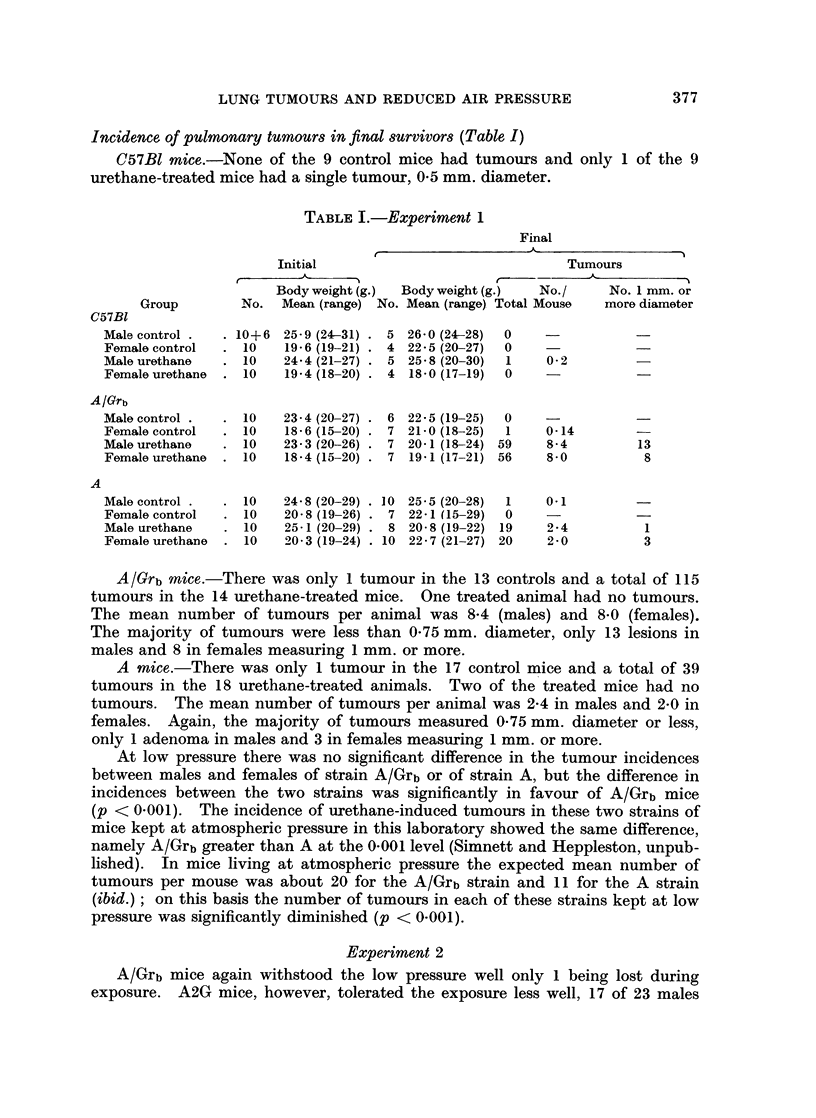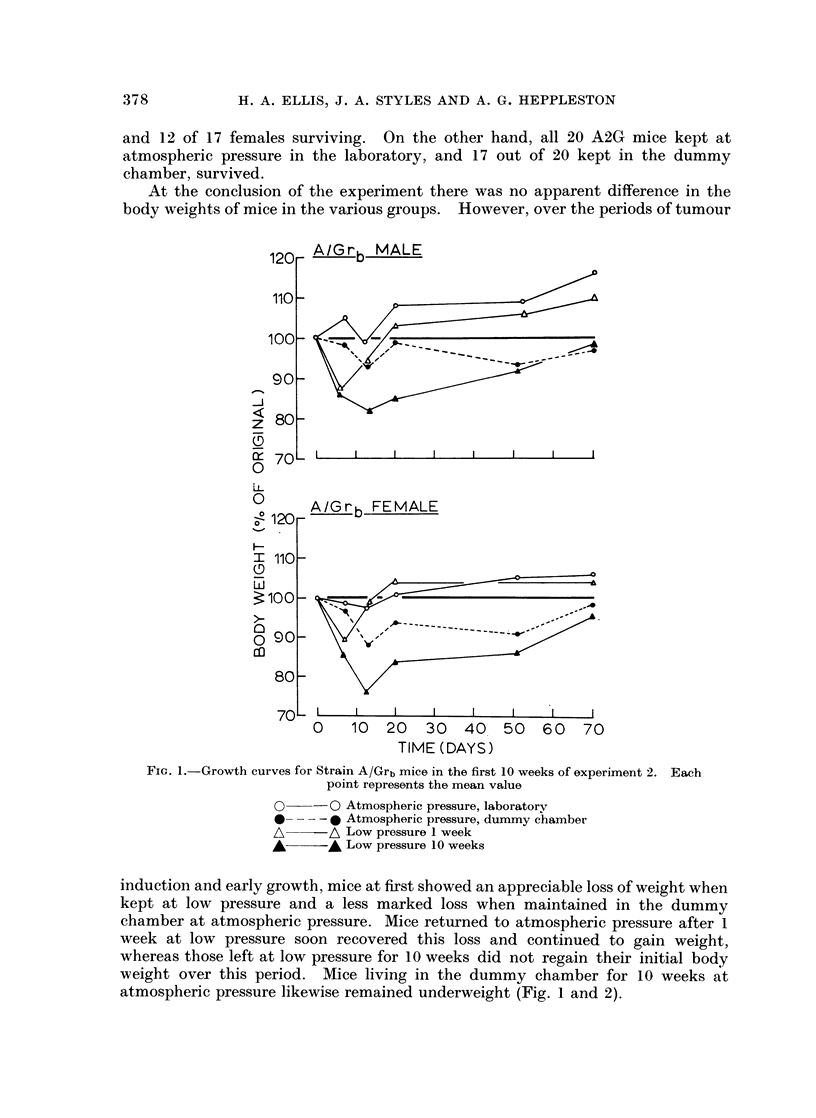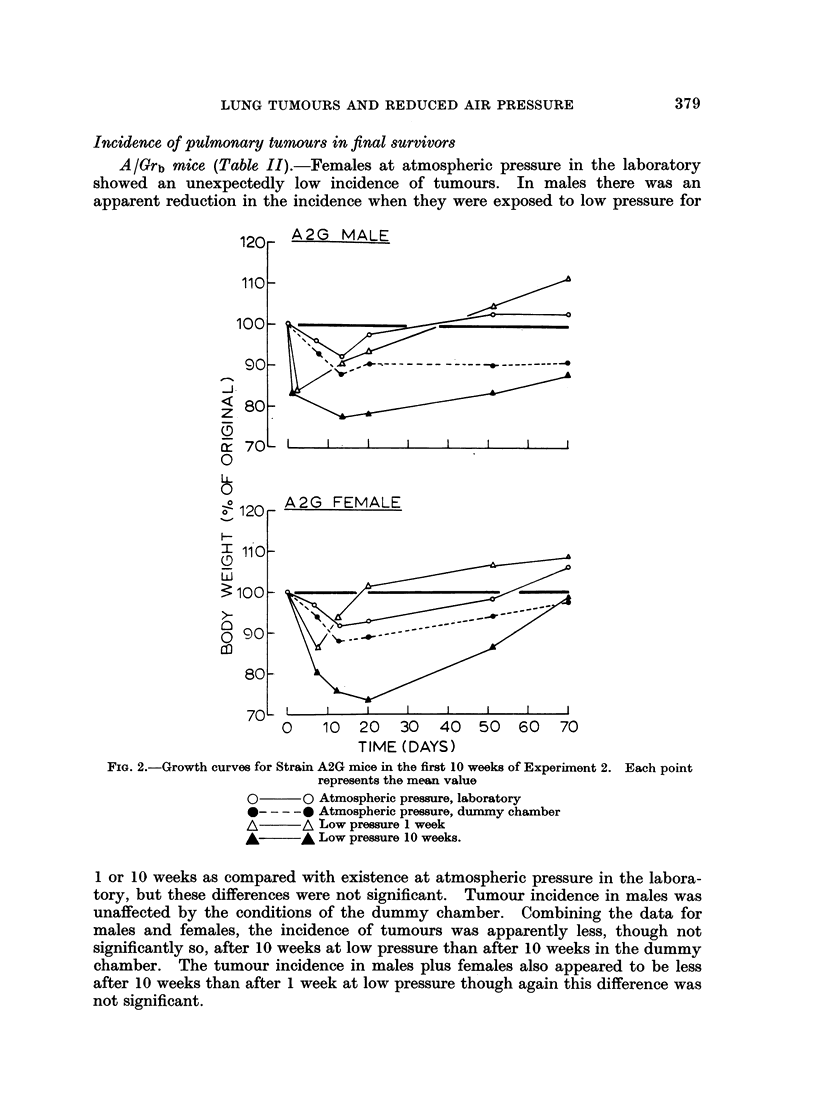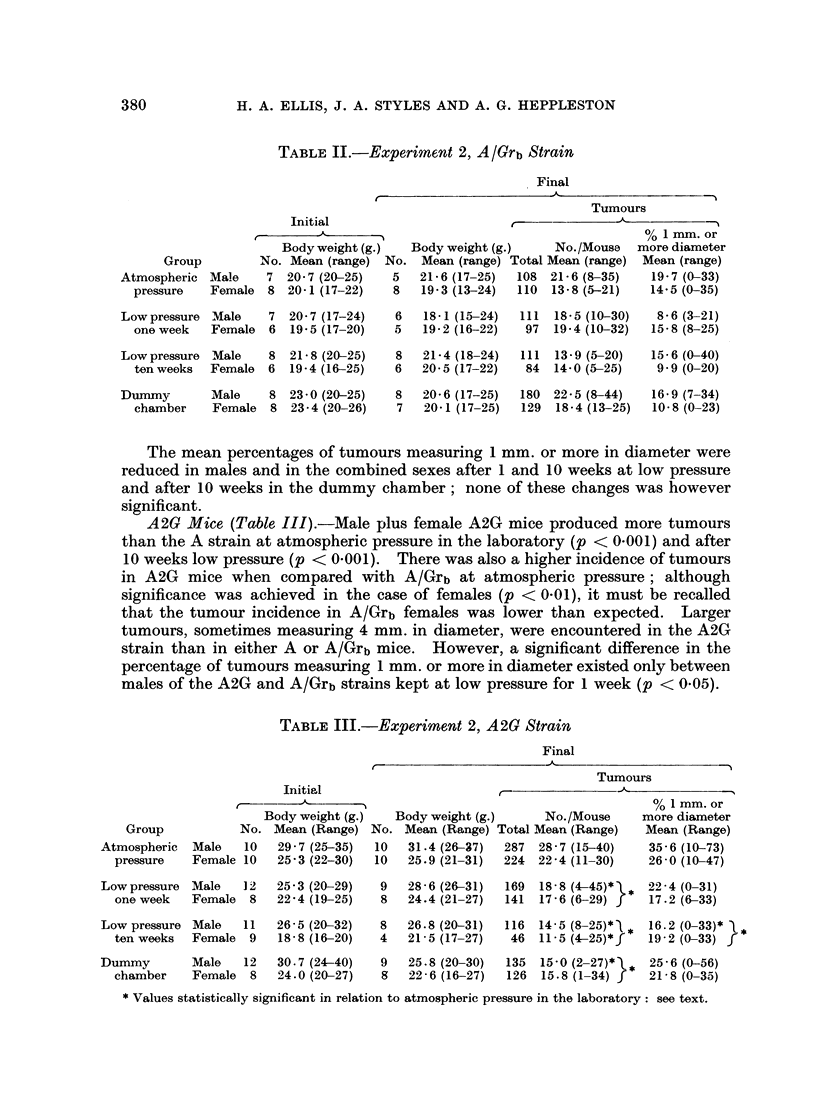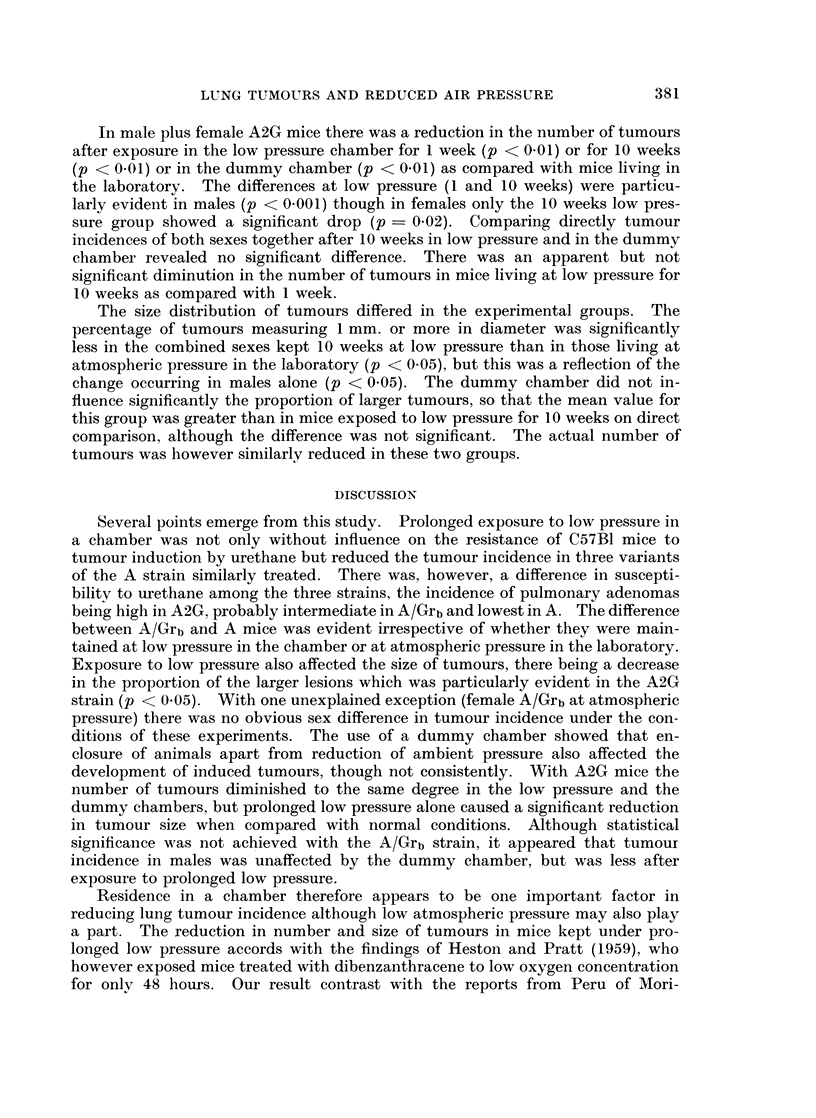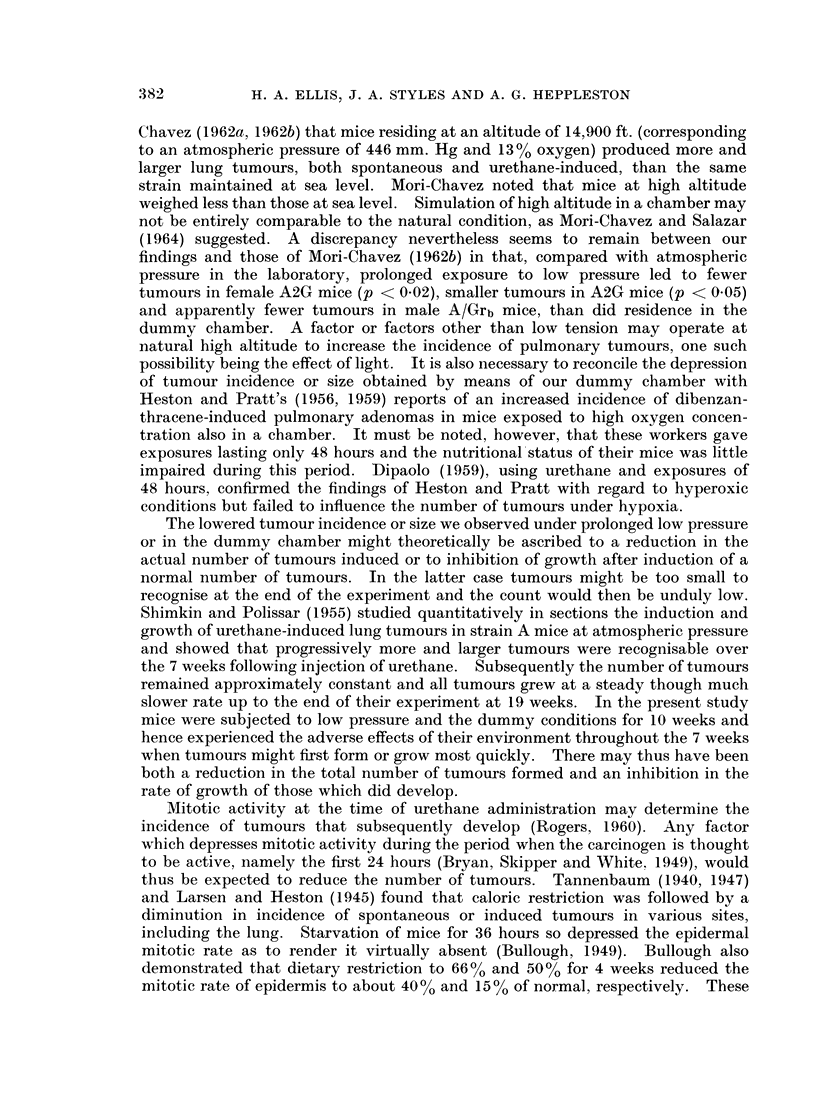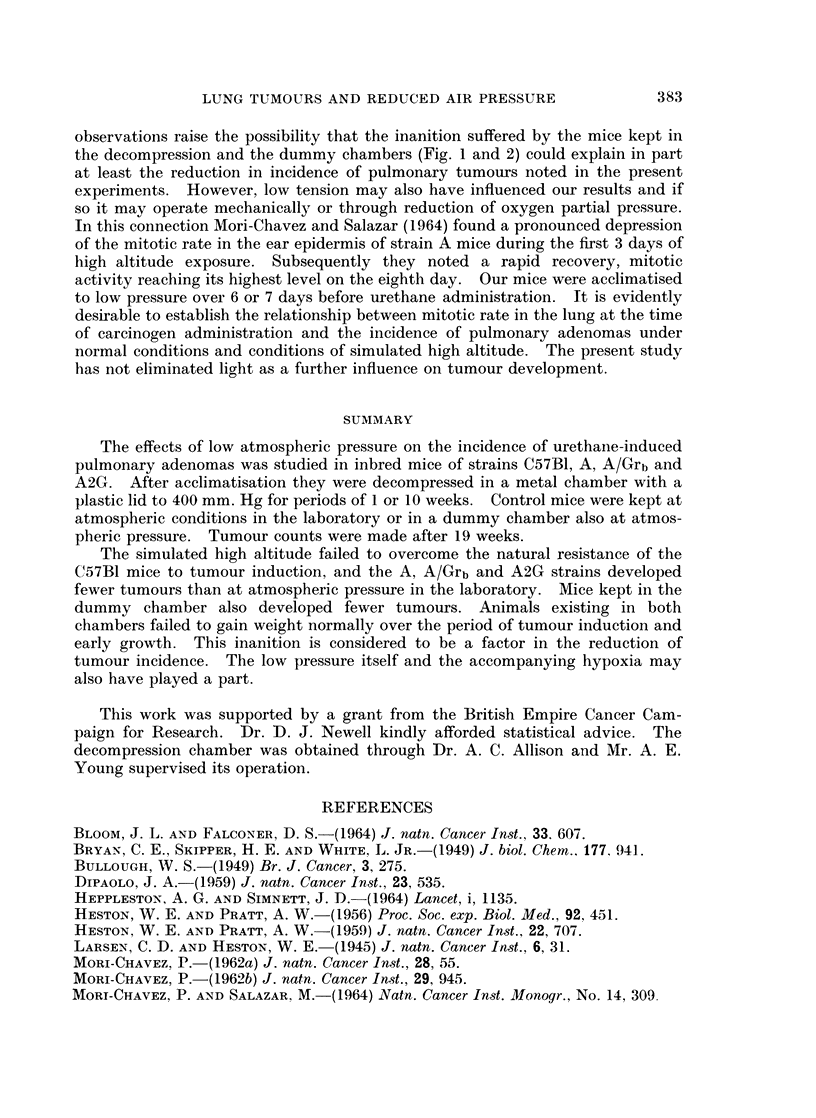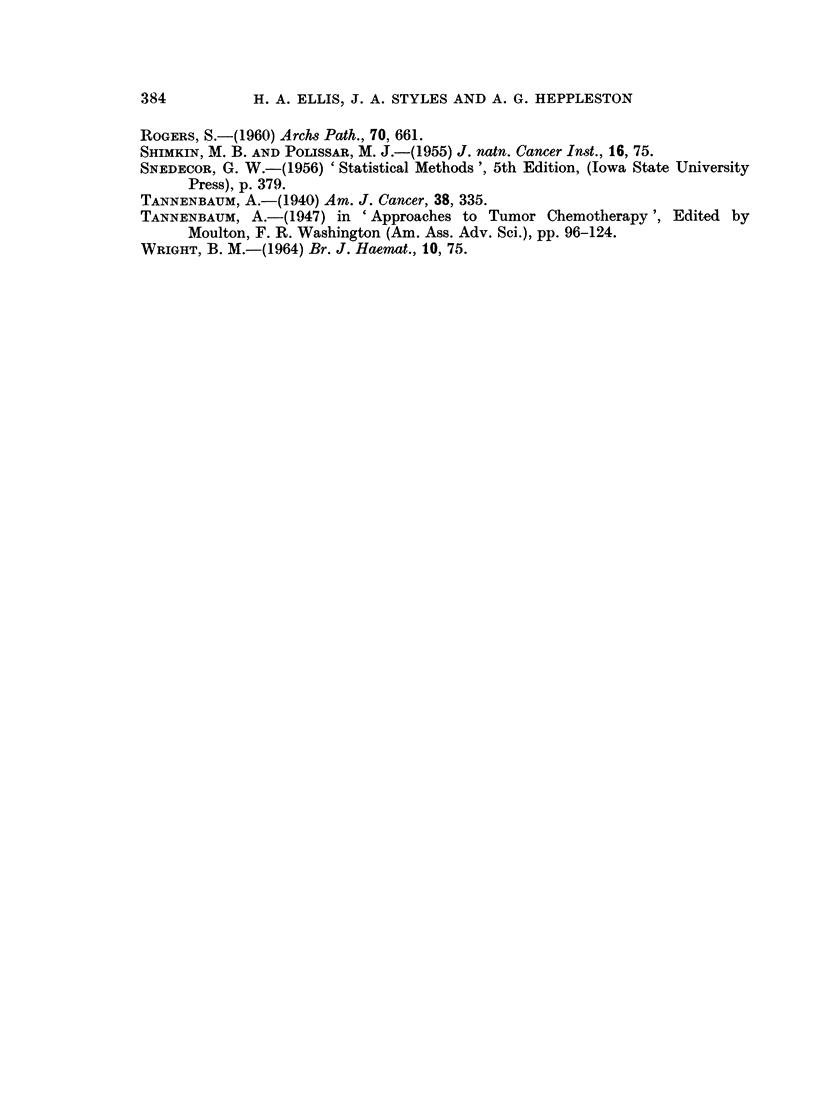# Incidence of lung tumours induced by urethane in mice exposed to reduced atmospheric pressure.

**DOI:** 10.1038/bjc.1966.45

**Published:** 1966-06

**Authors:** H. A. Ellis, J. A. Styles, A. G. Heppleston


### INCIDENCE OF LUNG TUMOURS INDUCED BY URETHANE IN MICE EXPOSED TO REDUCED ATMOSPHERIC PRESSURE

H. A. ELLIS, J. A. STYLES AND A. G. HEPPLESTON

*From the Department of Pathology, University of Newcastle upon Tyne*

Received for publication February 2, 1966

INBRED mice of strain A are well known to possess a high incidence of pulmonary adenomas whether spontaneous or induced, whereas strain C57Bl mice show a very low incidence, which according to Bloom and Falconer (1964) is genetically determined. In attempting to overcome this natural resistance of C57Bl mice to tumour development several observations led us to vary the oxygen tension in which mice were maintained. Heston and Pratt (1956, 1959) employed high or low oxygen tensions in a chamber for two days and found respectively increased or diminished incidences of dibenzanthracene-induced lung tumours in strain A mice. On the other hand, Mori-Chavez (1962*b*) observed an increase in the number and in the size of urethane-induced tumours in strain A mice maintained at a natural high altitude for eight or more months, and Heppleston and Simnett (1964) found that elevated oxygen tension produced deleterious effects on lung tissue and pulmonary adenomas from high incidence strains maintained in organ culture. Strains of mice susceptible and resistant to tumour induction by urethane were therefore exposed for prolonged periods to low atmospheric pressure by means of a decompression chamber.

Not only did this procedure fail to overcome the resistance of C57Bl mice to tumour induction, but mice of strains A and A/$Gr_b$ produced fewer lung tumours than expected on the basis of previous observations carried out in the normal atmosphere (Simnett and Heppleston, unpublished). Consequently it became necessary to study the effect of living in the chamber apart from the response to pressure reduction. Prolonged residence in a chamber at atmospheric pressure proved to be a factor in reducing the incidence of induced pulmonary tumours in susceptible strains of mice.

### MATERIALS AND METHODS

*The decompression chamber* was of the type described by Wright (1964), being made of metal with a plastic cover. After a preliminary period of acclimatisation over 6 or 7 days, during which the tension was lowered on alternate days by about 100 mm. mercury, the pressure was maintained at approximately one half an atmosphere (400 mm. mercury). The reverse procedure was adopted over a 5-day period when the mice were finally returned to atmospheric pressure.

*The dummy chamber* resembled the decompression chamber in size and shape, and both stood in the same small room. The dummy chamber was open at the top and air was extracted continuously from its base at a rate comparable to the flow through the decompression chamber. Temperature, humidity and light were therefore similar.

*Urethane.*—After acclimatisation urethane was injected intraperitoneally as a 4% aqueous solution in a single dose equivalent to 1 mg. per g. body weight. The mice were returned to the decompression chamber within an hour.

*General methods.*—All mice had free access to water and a cube diet (Spillers' breeding sow cubes with added vitamins A and D), and were weighed regularly. Food and water were usually replenished in the decompression chamber on alternate days, the period at atmospheric pressure being 5 to 15 minutes.

Survivors were killed by cervical fracture and the lungs fixed by inflation with formol-saline. The lobes were then separated and the tumours counted and measured under water against squared graph gaper using a dissecting microscope at $\times 5$. The nature of any doubtful lesions was established by histological examination.

*Experiment* 1.—After acclimatisation, 10 male and 10 female mice of each of the three strains C57Bl, A and $A/Gr_b$, aged between 3 and 5 months, were given urethane. Control mice in the same numbers of each sex did not receive urethane. All animals were kept in the decompression chamber for 10 weeks and then transferred to atmospheric pressure for 9 weeks before tumour counts were made. The C57Bl strain showed a poor tolerance to low pressure and, to make up for deaths, a further 6 male controls were added after 7 weeks and given the same treatment as the others. The total number of mice used was 126 (Table I).

*Experiment* 2.—Strains $A/Gr_b$ (58 mice) and A2G (80 mice) were used ; all animals were 3 to 5 months old and all were injected with urethane. Of the $A/Gr_b$ strain, 15 mice were kept at atmospheric pressure in the laboratory, 16 at atmospheric pressure in the dummy chamber for 10 weeks, and 27 in the low pressure chamber. Thirteen of the latter were transferred to atmospheric pressure after 1 week, the remaining 14 staying in the chamber for 10 weeks before transfer. All mice were killed at 19 weeks (Table II). The procedure with the A2G strain was similar, 20 mice serving as laboratory controls, 20 being kept at atmospheric pressure in the dummy chamber for 10 weeks followed by 9 weeks in the laboratory, and 40 in the low pressure chamber, half for 1 week and half for 10 weeks before completion of the 19 week study period at atmospheric pressure (Table III).

The statistical significance of differences in tumour incidence and size between the various groups had to be assessed by $2 \times 2$ factorial analysis for disproportionate sub-class numbers when the sexes were combined (Snedecor, 1956).

Random samples of different ages from the stock C57Bl, A, $A/Gr_b$ and A2G strains were shown to be free from polyoma virus infection, through the courtesy of Professor Michael Stoker.

### RESULTS

### *Experiment* 1

During the 10 week period of low pressure there were 35 deaths. Only 23 of 46 C57Bl mice survived this phase but there were few deaths after return to atmospheric pressure. Cannibalism precluded post-mortem examination in some mice ; others died from intercurrent infections. Compared with laboratory mice, those in the chamber often failed to thrive. Because the numbers of survivors differed between groups and because the body weight changes were rather variable, further attention was devoted to this aspect in the second experiment.

*Incidence of pulmonary tumours in final survivors (Table I)*

*C57Bl mice.*—None of the 9 control mice had tumours and only 1 of the 9 urethane-treated mice had a single tumour, 0·5 mm. diameter.

TABLE I.—*Experiment* 1

| | Initial | | Final | | | | |
|---|---|---|---|---|---|---|---|
| | | | | | Tumours | | |
| Group | No. | Body weight (g.) Mean (range) | No. | Body weight (g.) Mean (range) | Total | No./ Mouse | No. 1 mm. or more diameter |
| *C57Bl* | | | | | | | |
| Male control | 10+6 | 25·9 (24–31) | 5 | 26·0 (24–28) | 0 | — | — |
| Female control | 10 | 19·6 (19–21) | 4 | 22·5 (20–27) | 0 | — | — |
| Male urethane | 10 | 24·4 (21–27) | 5 | 25·8 (20–30) | 1 | 0·2 | — |
| Female urethane | 10 | 19·4 (18–20) | 4 | 18·0 (17–19) | 0 | — | — |
| *A/Gr*b | | | | | | | |
| Male control | 10 | 23·4 (20–27) | 6 | 22·5 (19–25) | 0 | — | — |
| Female control | 10 | 18·6 (15–20) | 7 | 21·0 (18–25) | 1 | 0·14 | — |
| Male urethane | 10 | 23·3 (20–26) | 7 | 20·1 (18–24) | 59 | 8·4 | 13 |
| Female urethane | 10 | 18·4 (15–20) | 7 | 19·1 (17–21) | 56 | 8·0 | 8 |
| *A* | | | | | | | |
| Male control | 10 | 24·8 (20–29) | 10 | 25·5 (20–28) | 1 | 0·1 | — |
| Female control | 10 | 20·8 (19–26) | 7 | 22·1 (15–29) | 0 | — | — |
| Male urethane | 10 | 25·1 (20–29) | 8 | 20·8 (19–22) | 19 | 2·4 | 1 |
| Female urethane | 10 | 20·3 (19–24) | 10 | 22·7 (21–27) | 20 | 2·0 | 3 |

*A/Gr*b *mice.*—There was only 1 tumour in the 13 controls and a total of 115 tumours in the 14 urethane-treated mice. One treated animal had no tumours. The mean number of tumours per animal was 8·4 (males) and 8·0 (females). The majority of tumours were less than 0·75 mm. diameter, only 13 lesions in males and 8 in females measuring 1 mm. or more.

*A mice.*—There was only 1 tumour in the 17 control mice and a total of 39 tumours in the 18 urethane-treated animals. Two of the treated mice had no tumours. The mean number of tumours per animal was 2·4 in males and 2·0 in females. Again, the majority of tumours measured 0·75 mm. diameter or less, only 1 adenoma in males and 3 in females measuring 1 mm. or more.

At low pressure there was no significant difference in the tumour incidences between males and females of strain A/Grb or of strain A, but the difference in incidences between the two strains was significantly in favour of A/Grb mice ($p < 0{\cdot}001$). The incidence of urethane-induced tumours in these two strains of mice kept at atmospheric pressure in this laboratory showed the same difference, namely A/Grb greater than A at the 0·001 level (Simnett and Heppleston, unpublished). In mice living at atmospheric pressure the expected mean number of tumours per mouse was about 20 for the A/Grb strain and 11 for the A strain (*ibid.*) ; on this basis the number of tumours in each of these strains kept at low pressure was significantly diminished ($p < 0{\cdot}001$).

### *Experiment* 2

A/Grb mice again withstood the low pressure well only 1 being lost during exposure. A2G mice, however, tolerated the exposure less well, 17 of 23 males and 12 of 17 females surviving. On the other hand, all 20 A2G mice kept at atmospheric pressure in the laboratory, and 17 out of 20 kept in the dummy chamber, survived.

At the conclusion of the experiment there was no apparent difference in the body weights of mice in the various groups. However, over the periods of tumour

Fig. 1.—Growth curves for Strain A/Gr$_b$ mice in the first 10 weeks of experiment 2. Each point represents the mean value

○———○ Atmospheric pressure, laboratory

●- - - -● Atmospheric pressure, dummy chamber

△———△ Low pressure 1 week

▲———▲ Low pressure 10 weeks induction and early growth, mice at first showed an appreciable loss of weight when kept at low pressure and a less marked loss when maintained in the dummy chamber at atmospheric pressure. Mice returned to atmospheric pressure after 1 week at low pressure soon recovered this loss and continued to gain weight, whereas those left at low pressure for 10 weeks did not regain their initial body weight over this period. Mice living in the dummy chamber for 10 weeks at atmospheric pressure likewise remained underweight (Fig. 1 and 2).

*Incidence of pulmonary tumours in final survivors*

*A/Gr$_b$ mice (Table II)*.—Females at atmospheric pressure in the laboratory showed an unexpectedly low incidence of tumours. In males there was an apparent reduction in the incidence when they were exposed to low pressure for

FIG. 2.—Growth curves for Strain A2G mice in the first 10 weeks of Experiment 2. Each point represents the mean value

○———○ Atmospheric pressure, laboratory

●- - - -● Atmospheric pressure, dummy chamber

△———△ Low pressure 1 week

▲———▲ Low pressure 10 weeks.

1 or 10 weeks as compared with existence at atmospheric pressure in the laboratory, but these differences were not significant. Tumour incidence in males was unaffected by the conditions of the dummy chamber. Combining the data for males and females, the incidence of tumours was apparently less, though not significantly so, after 10 weeks at low pressure than after 10 weeks in the dummy chamber. The tumour incidence in males plus females also appeared to be less after 10 weeks than after 1 week at low pressure though again this difference was not significant.

TABLE II.—*Experiment 2, A/Gr$_b$ Strain*

| Group | | Initial No. | Initial Body weight (g.) Mean (range) | Final No. | Final Body weight (g.) Mean (range) | Final Tumours Total | Final Tumours No./Mouse Mean (range) | Final Tumours % 1 mm. or more diameter Mean (range) |
|---|---|---|---|---|---|---|---|---|
| Atmospheric pressure | Male | 7 | 20·7 (20–25) | 5 | 21·6 (17–25) | 108 | 21·6 (8–35) | 19·7 (0–33) |
| | Female | 8 | 20·1 (17–22) | 8 | 19·3 (13–24) | 110 | 13·8 (5–21) | 14·5 (0–35) |
| Low pressure one week | Male | 7 | 20·7 (17–24) | 6 | 18·1 (15–24) | 111 | 18·5 (10–30) | 8·6 (3–21) |
| | Female | 6 | 19·5 (17–20) | 5 | 19·2 (16–22) | 97 | 19·4 (10–32) | 15·8 (8–25) |
| Low pressure ten weeks | Male | 8 | 21·8 (20–25) | 8 | 21·4 (18–24) | 111 | 13·9 (5–20) | 15·6 (0–40) |
| | Female | 6 | 19·4 (16–25) | 6 | 20·5 (17–22) | 84 | 14·0 (5–25) | 9·9 (0–20) |
| Dummy chamber | Male | 8 | 23·0 (20–25) | 8 | 20·6 (17–25) | 180 | 22·5 (8–44) | 16·9 (7–34) |
| | Female | 8 | 23·4 (20–26) | 7 | 20·1 (17–25) | 129 | 18·4 (13–25) | 10·8 (0–23) |

The mean percentages of tumours measuring 1 mm. or more in diameter were reduced in males and in the combined sexes after 1 and 10 weeks at low pressure and after 10 weeks in the dummy chamber ; none of these changes was however significant.

*A2G Mice* (*Table III*).—Male plus female A2G mice produced more tumours than the A strain at atmospheric pressure in the laboratory ($p < 0{\cdot}001$) and after 10 weeks low pressure ($p < 0{\cdot}001$). There was also a higher incidence of tumours in A2G mice when compared with A/Gr$_b$ at atmospheric pressure ; although significance was achieved in the case of females ($p < 0{\cdot}01$), it must be recalled that the tumour incidence in A/Gr$_b$ females was lower than expected. Larger tumours, sometimes measuring 4 mm. in diameter, were encountered in the A2G strain than in either A or A/Gr$_b$ mice. However, a significant difference in the percentage of tumours measuring 1 mm. or more in diameter existed only between males of the A2G and A/Gr$_b$ strains kept at low pressure for 1 week ($p < 0{\cdot}05$).

TABLE III.—*Experiment 2, A2G Strain*

| Group | | Initial No. | Initial Body weight (g.) Mean (Range) | Final No. | Final Body weight (g.) Mean (Range) | Final Tumours Total | Final Tumours No./Mouse Mean (Range) | Final Tumours % 1 mm. or more diameter Mean (Range) |
|---|---|---|---|---|---|---|---|---|
| Atmospheric pressure | Male | 10 | 29·7 (25–35) | 10 | 31.4 (26–37) | 287 | 28·7 (15–40) | 35·6 (10–73) |
| | Female | 10 | 25·3 (22–30) | 10 | 25.9 (21–31) | 224 | 22·4 (11–30) | 26·0 (10–47) |
| Low pressure one week | Male | 12 | 25·3 (20–29) | 9 | 28·6 (26–31) | 169 | 18·8 (4–45)* } * | 22·4 (0–31) |
| | Female | 8 | 22·4 (19–25) | 8 | 24.4 (21–27) | 141 | 17·6 (6–29) } | 17.2 (6–33) |
| Low pressure ten weeks | Male | 11 | 26·5 (20–32) | 8 | 26.8 (20–31) | 116 | 14·5 (8–25)* } * | 16.2 (0–33)* } * |
| | Female | 9 | 18·8 (16–20) | 4 | 21·5 (17–27) | 46 | 11·5 (4–25)* } | 19·2 (0–33) } |
| Dummy chamber | Male | 12 | 30.7 (24–40) | 9 | 25.8 (20–30) | 135 | 15·0 (2–27)* } * | 25·6 (0–56) |
| | Female | 8 | 24.0 (20–27) | 8 | 22·6 (16–27) | 126 | 15.8 (1–34) } | 21·8 (0–35) |

* Values statistically significant in relation to atmospheric pressure in the laboratory : see text.

In male plus female A2G mice there was a reduction in the number of tumours after exposure in the low pressure chamber for 1 week ($p < 0{\cdot}01$) or for 10 weeks ($p < 0{\cdot}01$) or in the dummy chamber ($p < 0{\cdot}01$) as compared with mice living in the laboratory. The differences at low pressure (1 and 10 weeks) were particularly evident in males ($p < 0{\cdot}001$) though in females only the 10 weeks low pressure group showed a significant drop ($p = 0{\cdot}02$). Comparing directly tumour incidences of both sexes together after 10 weeks in low pressure and in the dummy chamber revealed no significant difference. There was an apparent but not significant diminution in the number of tumours in mice living at low pressure for 10 weeks as compared with 1 week.

The size distribution of tumours differed in the experimental groups. The percentage of tumours measuring 1 mm. or more in diameter was significantly less in the combined sexes kept 10 weeks at low pressure than in those living at atmospheric pressure in the laboratory ($p < 0{\cdot}05$), but this was a reflection of the change occurring in males alone ($p < 0{\cdot}05$). The dummy chamber did not influence significantly the proportion of larger tumours, so that the mean value for this group was greater than in mice exposed to low pressure for 10 weeks on direct comparison, although the difference was not significant. The actual number of tumours was however similarly reduced in these two groups.

### DISCUSSION

Several points emerge from this study. Prolonged exposure to low pressure in a chamber was not only without influence on the resistance of C57Bl mice to tumour induction by urethane but reduced the tumour incidence in three variants of the A strain similarly treated. There was, however, a difference in susceptibility to urethane among the three strains, the incidence of pulmonary adenomas being high in A2G, probably intermediate in $A/Gr_b$ and lowest in A. The difference between $A/Gr_b$ and A mice was evident irrespective of whether they were maintained at low pressure in the chamber or at atmospheric pressure in the laboratory. Exposure to low pressure also affected the size of tumours, there being a decrease in the proportion of the larger lesions which was particularly evident in the A2G strain ($p < 0{\cdot}05$). With one unexplained exception (female $A/Gr_b$ at atmospheric pressure) there was no obvious sex difference in tumour incidence under the conditions of these experiments. The use of a dummy chamber showed that enclosure of animals apart from reduction of ambient pressure also affected the development of induced tumours, though not consistently. With A2G mice the number of tumours diminished to the same degree in the low pressure and the dummy chambers, but prolonged low pressure alone caused a significant reduction in tumour size when compared with normal conditions. Although statistical significance was not achieved with the $A/Gr_b$ strain, it appeared that tumour incidence in males was unaffected by the dummy chamber, but was less after exposure to prolonged low pressure.

Residence in a chamber therefore appears to be one important factor in reducing lung tumour incidence although low atmospheric pressure may also play a part. The reduction in number and size of tumours in mice kept under prolonged low pressure accords with the findings of Heston and Pratt (1959), who however exposed mice treated with dibenzanthracene to low oxygen concentration for only 48 hours. Our result contrast with the reports from Peru of Mori-

Chavez (1962*a*, 1962*b*) that mice residing at an altitude of 14,900 ft. (corresponding to an atmospheric pressure of 446 mm. Hg and 13% oxygen) produced more and larger lung tumours, both spontaneous and urethane-induced, than the same strain maintained at sea level. Mori-Chavez noted that mice at high altitude weighed less than those at sea level. Simulation of high altitude in a chamber may not be entirely comparable to the natural condition, as Mori-Chavez and Salazar (1964) suggested. A discrepancy nevertheless seems to remain between our findings and those of Mori-Chavez (1962*b*) in that, compared with atmospheric pressure in the laboratory, prolonged exposure to low pressure led to fewer tumours in female A2G mice ($p < 0{\cdot}02$), smaller tumours in A2G mice ($p < 0{\cdot}05$) and apparently fewer tumours in male $A/Gr_b$ mice, than did residence in the dummy chamber. A factor or factors other than low tension may operate at natural high altitude to increase the incidence of pulmonary tumours, one such possibility being the effect of light. It is also necessary to reconcile the depression of tumour incidence or size obtained by means of our dummy chamber with Heston and Pratt's (1956, 1959) reports of an increased incidence of dibenzanthracene-induced pulmonary adenomas in mice exposed to high oxygen concentration also in a chamber. It must be noted, however, that these workers gave exposures lasting only 48 hours and the nutritional status of their mice was little impaired during this period. Dipaolo (1959), using urethane and exposures of 48 hours, confirmed the findings of Heston and Pratt with regard to hyperoxic conditions but failed to influence the number of tumours under hypoxia.

The lowered tumour incidence or size we observed under prolonged low pressure or in the dummy chamber might theoretically be ascribed to a reduction in the actual number of tumours induced or to inhibition of growth after induction of a normal number of tumours. In the latter case tumours might be too small to recognise at the end of the experiment and the count would then be unduly low. Shimkin and Polissar (1955) studied quantitatively in sections the induction and growth of urethane-induced lung tumours in strain A mice at atmospheric pressure and showed that progressively more and larger tumours were recognisable over the 7 weeks following injection of urethane. Subsequently the number of tumours remained approximately constant and all tumours grew at a steady though much slower rate up to the end of their experiment at 19 weeks. In the present study mice were subjected to low pressure and the dummy conditions for 10 weeks and hence experienced the adverse effects of their environment throughout the 7 weeks when tumours might first form or grow most quickly. There may thus have been both a reduction in the total number of tumours formed and an inhibition in the rate of growth of those which did develop.

Mitotic activity at the time of urethane administration may determine the incidence of tumours that subsequently develop (Rogers, 1960). Any factor which depresses mitotic activity during the period when the carcinogen is thought to be active, namely the first 24 hours (Bryan, Skipper and White, 1949), would thus be expected to reduce the number of tumours. Tannenbaum (1940, 1947) and Larsen and Heston (1945) found that caloric restriction was followed by a diminution in incidence of spontaneous or induced tumours in various sites, including the lung. Starvation of mice for 36 hours so depressed the epidermal mitotic rate as to render it virtually absent (Bullough, 1949). Bullough also demonstrated that dietary restriction to 66% and 50% for 4 weeks reduced the mitotic rate of epidermis to about 40% and 15% of normal, respectively. These observations raise the possibility that the inanition suffered by the mice kept in the decompression and the dummy chambers (Fig. 1 and 2) could explain in part at least the reduction in incidence of pulmonary tumours noted in the present experiments. However, low tension may also have influenced our results and if so it may operate mechanically or through reduction of oxygen partial pressure. In this connection Mori-Chavez and Salazar (1964) found a pronounced depression of the mitotic rate in the ear epidermis of strain A mice during the first 3 days of high altitude exposure. Subsequently they noted a rapid recovery, mitotic activity reaching its highest level on the eighth day. Our mice were acclimatised to low pressure over 6 or 7 days before urethane administration. It is evidently desirable to establish the relationship between mitotic rate in the lung at the time of carcinogen administration and the incidence of pulmonary adenomas under normal conditions and conditions of simulated high altitude. The present study has not eliminated light as a further influence on tumour development.

### SUMMARY

The effects of low atmospheric pressure on the incidence of urethane-induced pulmonary adenomas was studied in inbred mice of strains C57Bl, A, A/$Gr_b$ and A2G. After acclimatisation they were decompressed in a metal chamber with a plastic lid to 400 mm. Hg for periods of 1 or 10 weeks. Control mice were kept at atmospheric conditions in the laboratory or in a dummy chamber also at atmospheric pressure. Tumour counts were made after 19 weeks.

The simulated high altitude failed to overcome the natural resistance of the C57Bl mice to tumour induction, and the A, A/$Gr_b$ and A2G strains developed fewer tumours than at atmospheric pressure in the laboratory. Mice kept in the dummy chamber also developed fewer tumours. Animals existing in both chambers failed to gain weight normally over the period of tumour induction and early growth. This inanition is considered to be a factor in the reduction of tumour incidence. The low pressure itself and the accompanying hypoxia may also have played a part.

This work was supported by a grant from the British Empire Cancer Campaign for Research. Dr. D. J. Newell kindly afforded statistical advice. The decompression chamber was obtained through Dr. A. C. Allison and Mr. A. E. Young supervised its operation.

### REFERENCES

Bloom, J. L. and Falconer, D. S.—(1964) *J. natn. Cancer Inst.*, **33**, 607.
Bryan, C. E., Skipper, H. E. and White, L. Jr.—(1949) *J. biol. Chem.*, **177**, 941.
Bullough, W. S.—(1949) *Br. J. Cancer*, **3**, 275.
Dipaolo, J. A.—(1959) *J. natn. Cancer Inst.*, **23**, 535.
Heppleston, A. G. and Simnett, J. D.—(1964) *Lancet*, i, 1135.
Heston, W. E. and Pratt, A. W.—(1956) *Proc. Soc. exp. Biol. Med.*, **92**, 451.
Heston, W. E. and Pratt, A. W.—(1959) *J. natn. Cancer Inst.*, **22**, 707.
Larsen, C. D. and Heston, W. E.—(1945) *J. natn. Cancer Inst.*, **6**, 31.
Mori-Chavez, P.—(1962*a*) *J. natn. Cancer Inst.*, **28**, 55.
Mori-Chavez, P.—(1962*b*) *J. natn. Cancer Inst.*, **29**, 945.
Mori-Chavez, P. and Salazar, M.—(1964) *Natn. Cancer Inst. Monogr.*, No. 14, 309.

ROGERS, S.—(1960) *Archs Path.*, **70**, 661.
SHIMKIN, M. B. AND POLISSAR, M. J.—(1955) *J. natn. Cancer Inst.*, **16**, 75.
SNEDECOR, G. W.—(1956) 'Statistical Methods', 5th Edition, (Iowa State University Press), p. 379.
TANNENBAUM, A.—(1940) *Am. J. Cancer*, **38**, 335.
TANNENBAUM, A.—(1947) in 'Approaches to Tumor Chemotherapy', Edited by Moulton, F. R. Washington (Am. Ass. Adv. Sci.), pp. 96–124.
WRIGHT, B. M.—(1964) *Br. J. Haemat.*, **10**, 75.